# Analysis of Differences in Metabolite Composition and Bioactivity of Black Mulberry Fruits from Four Production Regions in Xinjiang

**DOI:** 10.3390/foods15101747

**Published:** 2026-05-15

**Authors:** Shuang Liu, Ya Chen, Qian Tu, Shuai Liu, Xinyi Zhang, Chunlong Yuan, Jing Lei

**Affiliations:** 1 Turpan Experimental Station, Xinjiang Academy of Agricultural Sciences, Urumgi 830091, China; 2023051232@nwsuaf.edu.cn (S.L.); chenya1107@sina.com (Y.C.); 2 College of Enology, Northwest A&F University, Xianyang 712100, China; 2023060631@nwsuaf.edu.cn (Q.T.); ls18373928792@163.com (S.L.); 15686080965@163.com (X.Z.)

**Keywords:** aroma, color, bioactivity, differential metabolites

## Abstract

To elucidate the impacts of climatic and edaphic factors on the chemical composition and bioactivities of black mulberries, this study conducted a systematic comparative analysis of fruits sourced from four regions in Xinjiang: Turpan (T), Bayingolin Mongol Autonomous Prefecture (B), Hotan (H), and Kashgar (K). H exhibited the highest contents of total phenolics and total tannins, whereas T showed elevated levels of total flavonoids and flavan-3-ols. Notably, samples from B and H demonstrated superior antioxidant potential. Using UPLC-MS/MS, a total of 48 anthocyanin metabolites and 4 non-anthocyanin metabolites were identified in the mulberry fruits. Among these, 6 were region-specific compounds, and 8 were identified as differential metabolites. Furthermore, headspace solid-phase microextraction (HS-SPME) coupled with GC-MS/MS revealed 292 volatile metabolites, of which 15 were identified as differential metabolites based on OPLS-DA and relative odor activity value (rOAV) analyses. Metabolite profiling indicated that B possessed the greatest diversity of volatile metabolites, while T exhibited a remarkable richness in anthocyanin diversity. The observed regional variations in chemical constituents and metabolite profiles collectively accounted for the differences in antioxidant capacity and enzyme inhibitory activities among the black mulberry fruits. These findings provide a theoretical foundation for the regional characterization and targeted processing of black mulberries from the four production areas in Xinjiang.

## 1. Introduction

Mulberry (*Morus* spp.) belongs to the Moraceae family and is widely distributed across various regions of China. Common varieties include black mulberry (*Morus nigra* L.), white mulberry (*Morus alba* L.), red mulberry (*Morus rubra* L.), and golden mulberry (*Morus macroura* Miq.) [1]. Mulberry fruit quality is largely defined by appearance, aroma, chemical composition, and nutritional value. As a complex blend of diverse volatile compounds, aroma plays a key role in consumer perception and is co-regulated by factors such as cultivar, growing conditions, ecological environment, and genetic background [2]. Fresh mulberries are characterized by a complex aroma profile comprising green, acidic, sweet, woody, fruity, fresh, and fatty notes. These aroma characteristics are co-mediated by multiple aroma-active volatiles, including (E,Z)-2,6-nonadienal, β-ionone, hexanal, and (E)-2-nonenal [3]. The composition and content of volatile metabolites vary among mulberry fruits, leading to distinct aroma profiles. In addition to the sensory characteristics contributed by volatile aroma compounds, the phenolic compounds enriched in mulberries serve as a key material foundation for their nutritional quality and commercial value. Phenolic compounds not only enhance fruit color, aroma, and flavor complexity, but also improve nutritional value through their influence on fruit bioactivity. Previous research has explored the composition and nutritional value of secondary metabolites in mulberries, with particular emphasis on anthocyanins. As natural flavonoids localized in vacuoles, anthocyanins produce red, purple, and black hues and are the primary determinants of mulberry fruit color. The anthocyanin metabolites in mulberries mainly exist as anthocyanins, including delphinidin 3-O-diglucoside (Dp 3-O-dG), cyanidin 3-O-diglucoside (Cy 3-O-dG), and pelargonidin 3-O-diglucoside (Pg 3-O-dG) [4]. The phenolic content varies greatly among mulberry varieties. On a fresh weight basis, total phenolics range from 0.44 to 3.35 g GAE kg^−1^ in white mulberries, from 1.51 to 11.32 g GAE kg^−1^ in red mulberries, and from 0.95 to 10.99 g GAE kg^−1^ in black mulberries [5]. These differences are mainly due to genotype-dependent expression of physiological traits. However, even within the same variety, significant metabolic differences can arise under the influence of factors such as topography, terroir, temperature, and humidity when cultivated in different regions or under different growing conditions [6]. For example, in the same “DaShi” variety cultivated in Liyang, Jutong, Zhenjiang, Anji, and Chongqing, the content of cyanidin-3-O-glucoside was recorded at 2.056, 2.276, 2.094, 2.545, and 2.463 mg/g, respectively. The content of pelargonidin-3-O-rutinoside also varied markedly, ranging from 0.687 to 1.158 mg/g [7]. These metabolic differences not only affect the visual quality of mulberry fruits, but also significantly influence their bioactivity. Furthermore, studies have shown that black mulberries contain higher levels of polyphenolic compounds—such as cyanidin-3-O-glucoside and cyanidin-3-O-rutinoside—than white mulberries, thereby effectively inhibiting the production of reactive oxygen species (ROS), oxidative stress, and mitochondrial dysfunction [8]. Quercetin in mulberries possesses high nutritional value, as it activates adenosine monophosphate (AMP) and prevents lipid peroxidation, thereby promoting glucose transporter type 4 (GLUT4)—the key facilitator of glucose uptake in skeletal muscle, adipose tissue, and other peripheral tissues [9]. Given the combined effects of various metabolites, mulberry fruits from different production regions display distinct bioactivities. Consequently, identifying regional differences in phenolic compounds and volatile metabolites is essential for elucidating their bioactivity and advancing research on the nutritional value of mulberry fruits.

The Xinjiang Uygur Autonomous Region is known for its distinctive climate-arid conditions, low rainfall, large day–night temperature differences, and long sunshine hours, which makes it an ideal region for high-quality mulberry cultivation [10]. In sub-regions such as Turpan, Bazhou, Hotan, Kashgar, and Aksu, a rich diversity of premium mulberry varieties is grown, with black mulberries being the predominant type. Modern pharmacological research has revealed that black mulberry fruits are rich in bioactive compounds, including polyphenols, flavonoids, anthocyanins, and polysaccharides. Recognized as a medicinal and edible food, black mulberries offer a range of nutritional benefits, such as antioxidant, antidiabetic, anti-inflammatory, anti-obesity, and antitumor activities [11,12,13,14]. The distinct climatic conditions across different production regions in Xinjiang not only affect mulberry physiology and metabolism, but also critically shape the formation and expression of phenolic compounds and aroma characteristics, thereby influencing fruit bioactivity and nutritional value. Thus, identifying regional variations in the metabolite profiles of black mulberries is essential for a comprehensive assessment of fruit quality and bioactivity, and offers important guidance for the value-added processing of Xinjiang’s mulberry industry. Nevertheless, research on black mulberries from Xinjiang remains insufficiently comprehensive, especially regarding comparisons between sub-regions. Existing studies on mulberry fruit quality are often fragmented, focusing on isolated attributes such as aroma profiles or color expression. As a result, there is a notable lack of comparative investigations and holistic quality evaluations of black mulberry samples from different sub-regions within the same major production area.

To systematically assess the fruit quality of black mulberries, this study performed comprehensive qualitative and quantitative analyses of sugars, organic acids, phenolic compounds, anthocyanin metabolites, and volatile metabolites in black mulberries sourced from four production regions in Xinjiang. The antioxidant and hypoglycemic potentials of the fruits were evaluated based on their antioxidant capacity and enzyme inhibitory activity. Furthermore, correlation analyses between phenolic compounds/metabolites and bioactivity parameters were conducted to elucidate the differential bioactive contributions of various components within the black mulberry system. This study seeks to refine the quality evaluation system for black mulberries and to support the high-quality development of the mulberry industry, as well as the deep processing of value-added mulberry products in the production regions.

## 2. Materials and Methods

### 2.1. Materials and Chemicals

**Figure 1:**
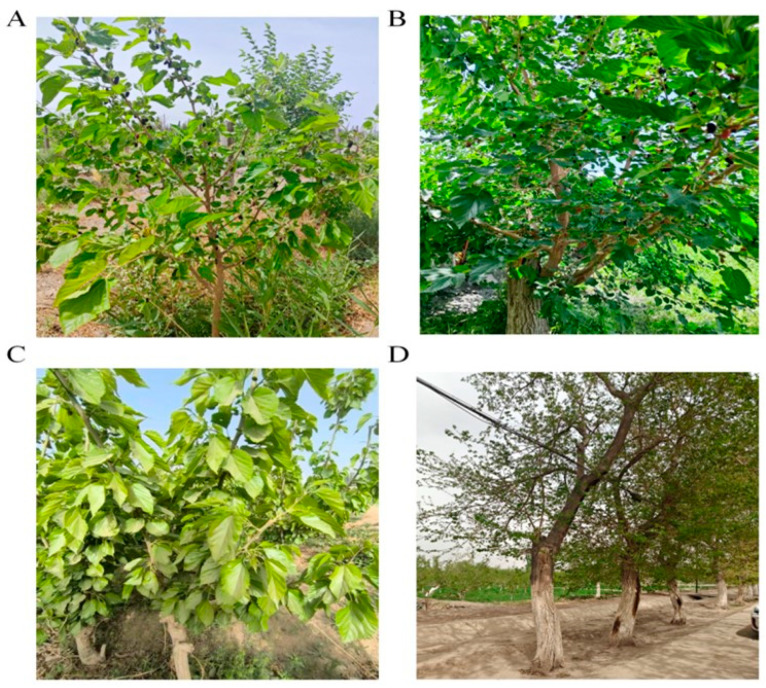
Contents of sugars, organic acids, and phenolic compounds in mulberry fruits from different production regions. Note: (**A**–**D**) represent the mulberry trees from the four production areas of ‘Turpan,’ ‘Bazhou,’ ‘Hotan,’ and ‘Kashgar’.

Materials: Fresh black mulberry (*Morus nigra* L.) fruits were collected in May 2024 from four regions: Turpan City, Korla City in the Bayingolin Mongol Autonomous Prefecture (hereinafter referred to as Bazhou), Hotan City, and Kashgar City in Xinjiang. Sampling information is shown in Table 1, and the data came from the National Earth System Science Data Center. At each sampling point, 1 kg of fresh mulberries was collected. Samples from the same origin were thoroughly mixed, and then random samples were taken for subsequent indicator measurements. The current status of mulberry trees at different sampling sites is shown in Figure 1.

### 2.2. Appearance Characteristics

Twenty fruits were randomly selected for quality and volume measurements. Fresh weight and dry weight were measured using a 0.001 g electronic balance, and volume was measured by the displacement of water in a graduated cylinder, with values averaged. Ten fruits were arbitrarily selected for appearance averaging; longitudinal diameter, transverse diameter, and peduncle length were measured using a digital Vernier caliper, and exterior color was measured using an NH310 colorimeter, recording L*, a*, and b* values, with results presented as the mean ± standard deviation. Fruits were randomly selected and three biological replicates were set, with 10 fruits per group. Soluble solid content of the fruits was measured using a digital refractometer, and fruit pH was measured using a pen-type pH meter (model: pH-10), with results represented as the mean. The reducing sugar content in mulberry was determined in accordance with the national standard of the People’s Republic of China (GB 5009.7–2016) [15].

### 2.3. Determination of Sugars and Organic Acids

Soluble sugars in mulberry fruits were analyzed using a high-performance liquid chromatography system (LC-20AT, Shimadzu, Kyoto, Japan) equipped with an Athena NH2-rp(II) column (4.6 mm × 250 mm, 5 μm) [16]. The column temperature was maintained at 40 °C. The mobile phase consisted of acetonitrile–water (70:30, *v*/*v*) at a flow rate of 1.0 mL/min. The injection volume was 10 μL.

Organic acid profiles were determined using a high-performance liquid chromatography system (LC-2030C Plus, Shimadzu) equipped with an Athena-C18 WP column (4.6 mm × 250 mm, 5 μm) [17]. The column temperature was set at 25 °C. The mobile phase comprised 0.02 mol/L potassium dihydrogen phosphate aqueous solution (pH 2.60) as phase A and methanol as phase B, applied in a gradient elution mode. The flow rate was 0.4 mL/min, and the injection volume was 10 μL.

### 2.4. Composition of Phenolic Substances

Fifty mulberry fruits that had been frozen at –80 °C for at least 48 h were promptly combined with liquid nitrogen under frozen conditions and ground into a fine powder using a mortar and pestle. The powdered sample was then lyophilized using a vacuum freeze dryer. An aliquot of 0.1 g of the freeze-dried powder was extracted with 2 mL of methanol–HCl solution (60% methanol containing 0.1% hydrochloric acid) under ultrasonication at 30 °C and 120 W for 30 min. The mixture was subsequently centrifuged at 5500× rpm for 10 min at 4 °C, and the supernatant was collected. This extraction procedure was repeated three times. The total phenolic content in mulberry samples was determined using the Folin–Ciocalteu colorimetric method at a wavelength of 765 nm [18]. Results were expressed as gallic acid equivalent (GAE) per gram of dry weight. Total flavonoid content was measured by aluminum chloride and sodium nitrite colorimetric assay at 510 nm, and the results were expressed as rutin equivalent (RE) per gram of dry weight [19]. Total anthocyanin content was determined using the pH differential method, with absorbance measured at 520 nm and 700 nm. Results were expressed as malvidin-3-O-glucoside (MA) equivalent per gram of dry weight [20]. Total flavanol content was analyzed using the p-DMACA–HCl method at 640 nm, and the results were expressed as (+)-catechin equivalent (CE) per gram of dry weight [21]. The total tannin content in mulberry samples was determined using the hydrochloric acid–vanillin assay. Absorbance was measured at 500 nm, and the results were expressed as (+)-catechin equivalent (CE) per gram of dry weight [22].

### 2.5. Volatile Compounds

Volatile metabolites in mulberry samples were analyzed using headspace solid-phase microextraction (HS-SPME) coupled with gas chromatography-tandem mass spectrometry (GC-MS/MS) [23,24]. A 120 μm DVB/CWR/PDMS fiber was inserted into the headspace vial containing the sample and maintained at 60 °C for 15 min to allow for adsorption. The fiber was then desorbed at 250 °C for 5 min in the GC injection port, followed by separation and identification via GC-MS/MS. The relative content of each volatile compound was calculated using the internal standard semi-quantification method according to the following formula:

X_i_ = (V_s_ × C_s_)/M × I_i_/I_s_ × 10^−3^

where:

X_i_ = content of compound in the sample (μg/g);

V_s_ = volume of the internal standard added (μL);

C_s_ = concentration of the internal standard (μg/mL);

M = mass of the sample (g);

I_s_ = peak area of the internal standard;

I_i_ = peak area of compound in the sample.

The relative odor activity value (rOAV) was calculated using the following formula:

rOAV_i_ = C_i_/T_i_

where:

rOAVi = relative odor activity value of compound;

Ci = relative content of compound;

Ti = odor threshold value of compound.

### 2.6. Anthocyanin Metabolite Analysis

Qualitative and quantitative analysis of anthocyanins in mulberry fruits was performed using ultra-performance liquid chromatography-tandem mass spectrometry (UPLC-MS/MS) [25]. Chromatographic separation was carried out on an ACQUITY BEH C18 column (1.7 µm, 2.1 mm × 100 mm). The mobile phase consisted of ultrapure water containing 0.5% formic acid (Phase A) and methanol containing 0.5% formic acid (Phase B).

Quantification of anthocyanin compounds was conducted using authentic reference standards, including Delphinidin-3-O-galactoside, Pelargonidin-3-O-glucoside, Pelargonidin-3,5-O-diglucoside, Peonidin-3-O-glucoside, Procyanidin B3, Procyanidin B1, Naringenin, Cyanidin-3-O-sambubioside, Cyanidin-3-O-arabinoside, Cyanidin-3-O-sophoroside, Cyanidin-3-O-(6-O-malonyl-beta-D-glucoside), Cyanidin-3-O-glucoside, Cyanidin-3-O-xyloside, Quercetin-3-O-glucoside, Malvidin-3-O-glucoside, Malvidin-3-O-galactoside, and Delphinidin-3-O-glucoside.

### 2.7. Antioxidant Activity

A 0.1 g aliquot of freeze-dried mulberry powder was mixed with 3 mL of 95% ethanol, followed by ultrasonic extraction at 30 °C for 30 min. The mixture was centrifuged at 5500 rpm for 20 min at 4 °C, and the supernatant was collected. The extraction procedure was repeated three times, and the supernatants were combined and used as the test solution for subsequent antioxidant activity assays.

A 5 mL aliquot of 7 mmol/L ABTS solution was mixed with 88 μL of 140 mmol/L potassium persulfate, and the mixture was allowed to react in the dark for 12 h [26]. The resulting ABTS^+^ working solution was diluted with 80% ethanol to an absorbance of approximately 0.7 at 734 nm. Subsequently, 2 mL of the ABTS working solution was mixed with 2 mL of sample extract and incubated in the dark for 5 min, after which the absorbance was measured at 734 nm. A sample control was prepared by mixing 2 mL of 80% ethanol with 2 mL of sample extract. A 2 mL aliquot of 0.4% DPPH–ethanol solution was mixed with 2 mL of the sample extract. The sample control consisted of an equal volume of anhydrous ethanol in place of the DPPH–ethanol solution, while the blank group used anhydrous ethanol instead of the sample extract. Absorbance was measured at 517 nm [27]. Hydroxyl radical scavenging activity was determined according to the method of others [28]. Briefly, 0.6 mL of the fivefold diluted sample extract was mixed with 2 mL of 9 mmol/L ferrous sulfate solution and 2 mL of 8.8 mmol/L hydrogen peroxide solution. The mixture was allowed to stand at room temperature for 10 min, after which 2 mL of 9 mmol/L salicylic acid–ethanol solution was added. The reaction was carried out at 37 °C for 30 min, and the absorbance was measured at 510 nm. A control sample was prepared by replacing the hydrogen peroxide solution with an equal volume of deionized water. The scavenging rate was calculated using the following formula:

Scavenging rate (%) = (1 − A_1_ − A_2_A_0_) × 100%

where:

A_1_ = absorbance of the sample group,

A_2_ = absorbance of the sample control group,

A_0_ = absorbance of the blank group.

### 2.8. Enzyme Inhibitory Activity

A 0.1 g aliquot of freeze-dried mulberry powder was mixed with 1 mL of phosphate buffer, vortexed thoroughly, and centrifuged at 10,000 rpm for 10 min at 4 °C. The supernatant was collected and kept on ice for subsequent enzyme inhibition assays. The inhibitory activity of the extract against α-amylase was determined following previously established methods [29]. Briefly, 50 μL of the sample was mixed with 50 μL of α-amylase solution (20 U/mL) and incubated at 37 °C for 15 min. Then, 1.25 mL of gelatinized starch solution (10 mg/mL) was added, and the mixture was incubated at 37 °C for another 15 min. The reaction was terminated by heating at 100 °C for 5 min, followed by centrifugation. An aliquot of 200 μL of the supernatant was mixed with 200 μL of PAHBAH reagent, heated in a boiling water bath for 5 min, and then diluted with 3.6 mL of distilled water. Absorbance was measured at 410 nm using a microplate reader. The sample control was prepared by replacing the enzyme solution with an equal volume of PBS, the blank group by replacing the sample with PBS, and the blank control by replacing both the sample and enzyme with PBS. The inhibitory effect of mulberry polysaccharides on α-glucosidase activity was evaluated according to the method of others [30]. α-Glucosidase (0.05 mg/mL) and 4-nitrophenyl-β-D-glucopyranoside (pNPG, 5 mmol/L) were prepared in 0.1 mol/L phosphate buffer (pH 6.8). A 0.05 mL aliquot of the polysaccharide solution was mixed with 0.1 mL of enzyme solution and pre-incubated at 37.5 °C for 10 min. Then, 0.1 mL of pNPG solution was added, and the mixture was incubated at 37.5 °C for an additional 10 min. The reaction was terminated by adding 1 mL of 1 mol/L sodium carbonate solution, and absorbance was measured at 405 nm. Inhibition of pancreatic lipase by mulberry polysaccharides was assessed following the method of Lu et al. [31]. Briefly, 50 μL of the polysaccharide sample (1 mg/mL) was mixed with 200 μL of pancreatic lipase solution and incubated at 37 °C for 15 min. Then, 50 μL of 0.4% p-nitrophenyl laurate (pNP laurate) was added, and the mixture was incubated at 37 °C for 45 min. The reaction was terminated by adding 300 μL of 5 mmol/L sodium acetate buffer (pH 5.0), followed by centrifugation at 10,000 rpm for 5 min. Absorbance of the supernatant was measured at 405 nm. The inhibition rate (%) was calculated using the following formula:

Inhibition rate (%) = [1 − A_1_ − A_2_A_0_ − A_0_′] × 100%

where:

A_1_ = absorbance of the sample group,

A_2_ = absorbance of the sample control group,

A_0_ = absorbance of the blank group,

A_0_′ = absorbance of the blank control group.

### 2.9. Statistical Analysis

Data processing and significance analysis were conducted using SPSS V20 (IBM, New York, NY, USA). Tukey’s test was employed for statistical analysis at a significance level of *p* < 0.05. Experiments were repeated at least three times, with results presented as the mean ± standard deviation. Column plots and correlation heat maps were generated using Origin 2021 (OriginLab Corporation, Northampton, MA, USA).

## 3. Results and Discussion

### 3.1. Fruit Appearance Quality and Basic Physicochemical Properties

Influenced by their respective growing conditions, black mulberry fruits exhibited distinct regional variations across the four production areas. As shown in Table 2, fruit volume ranged from 1.57 to 6.98 cm^3^, and fresh weight ranged from 1.26 to 7.98 g, which were generally consistent with previously reported values [32]. The trends in fruit volume and fresh weight followed a similar pattern: K < T < H < B, with no significant differences observed between the T and K samples, while significant differences were found between the B and H samples. Based on the dry weight of twenty fruits, the order was T < H < K < B. No significant differences in dry weight were observed between K and B or between K and H; however, all three regions differed significantly from T, likely due to variations in fruit water content. Among the four regional samples, T and H exhibited similar transverse and longitudinal diameters, whereas B and K showed the greatest morphological differences. Fruit color, determined by L, a, and b* values, varied with factors such as temperature and sunlight exposure. All four regional fruits displayed a purplish-red coloration. Notably, fruits from T appeared darker (more blackish), while those from B exhibited stronger red and blue tones; no significant color differences were observed between H and K. Total soluble solids (TSS) content ranged from 8.14 °Brix to 20.93 °Brix, with significant differences detected between B and H, possibly attributable to variations in sunshine duration during the fruit growth period. These TSS values are relatively higher than the previously reported range of 5.66 to 15.62 °Brix, which may be attributed to differences in production region, cultivar, or other influencing factors [33].

### 3.2. Composition of Sugars and Organic Acids in Mulberry Fruits

**Figure 2:**
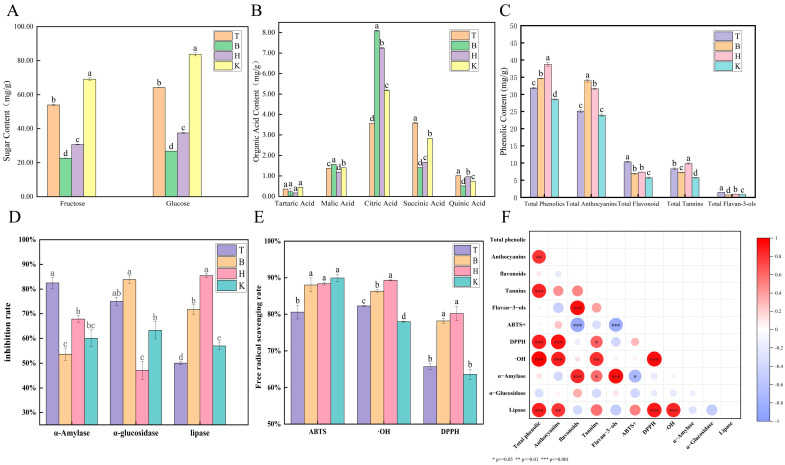
Contents of sugars, organic acids, and phenolic compounds in mulberry fruits from different production regions. Note: (**A**,**B**) Sugar and acid content, results calculated based on fresh fruit weight (FW); (**C**) phenolic content, calculated based on dry weight (DW); (**D**) enzyme activity inhibition rate; (**E**) free radical scavenging rate; (**F**) correlation analysis. Bars with a different letter are significantly different according to Duncan’s multiple range test at *p* < 0.05 level.

The primary soluble sugars in mulberry fruits were fructose and glucose (Figure 2A), with distinct variations observed among the four production regions. Among the four regions, sample K had the highest fructose and glucose contents (83.75 mg/g and 69.14 mg/g, respectively), presumably due to the large diurnal temperature variation that favors photosynthetic product accumulation. Sample T ranked second (53.99 mg/g and 64.26 mg/g, respectively), which may be attributed to the longest sunshine duration (up to 2788 h) and abundant thermal resources in Turpan, promoting rapid sugar synthesis. Sample B showed the lowest fructose and glucose contents (22.62 mg/g and 26.79 mg/g, respectively).

Tartaric acid, malic acid, citric acid, succinic acid, and quinic acid were identified as the major organic acids in mulberry fruits (Figure 2B). Citric acid, malic acid, and succinic acid—important intermediates of the tricarboxylic acid (TCA) cycle—serve as key biochemical indicators of organic acid metabolism in plants. Quinic acid functions as a core carbon skeleton donor for the biosynthesis of phenolic compounds [34]. Overall, the organic acid profiles of mulberry fruits from the four regions exhibited significant regional differences, which may be attributable to variations in growing conditions and associated metabolic regulation. Citric acid was the most abundant organic acid in samples from Bazhou, Hotan, and Kashgar, at 8.09, 7.25, and 5.17 mg/g, respectively. This is likely due to the higher elevation (900–1400 m) and relatively lower temperatures in these areas, which may reduce citric acid degradation and favor its accumulation in the fruit. In sample T, succinic acid content (3.59 mg/g) was similar to that of citric acid (3.57 mg/g), whereas lower levels were found in samples B (1.41 mg/g) and H (1.67 mg/g). This is probably because the high heat and strong sunlight in Turpan promote an active tricarboxylic acid (TCA) cycle, resulting in marked succinic acid accumulation. Malic acid concentrations were similar across all four regions, ranging from 1.18 to 1.56 mg/g. Quinic acid content showed marked regional variation, with higher levels observed in T (1.02 mg/g) and H (0.96 mg/g), and lower levels in B (0.51 mg/g) and K (0.75 mg/g). Tartaric acid exhibited the lowest relative content among the five organic acids and showed no significant differences across the four black mulberry-producing regions.

### 3.3. Composition of Phenolic Compounds in Mulberry Fruits

Phenolic compounds play a vital role in determining the color, flavor, physicochemical properties, and nutritional value of mulberry fruits. In Figure 2C, Hotan mulberries (H) exhibited the highest contents of total phenolics (38.72 ± 0.63 mg/g) and total tannins (9.85 ± 0.22 mg/g), along with a relatively high anthocyanin content (31.62 ± 0.17 mg/g). This is likely attributable to factors such as high altitude, strong ultraviolet (UV) radiation, and large diurnal temperature variations, which may promote phenylpropane metabolism and enhance the accumulation of anthocyanins and tannins [35]. Turpan mulberries (T) showed significantly higher contents of total flavonoids (10.34 ± 0.14 mg/g) and flavan-3-ols (1.45 ± 0.02 mg/g) compared to the other production regions. This may be influenced by the extreme aridity and extreme high temperatures in the Turpan region. Extreme drought stress can activate phenylpropane metabolism and promote the biosynthesis of phenolic acids and flavonoids, thereby enhancing fruit resistance to drought stress [36]. In contrast, anthocyanin biosynthesis is dependent on low nighttime temperatures and adequate water availability, whereas extremely high temperatures accelerate their non-enzymatic hydrolysis and oxidative degradation [37]. Bazhou mulberries (B) had a higher anthocyanin content (33.95 ± 0.37 mg/g) but lower total tannin and flavan-3-ol contents, whereas Kashgar mulberries (K) exhibited relatively low phenolic levels overall. These differences may be related to non-standardized cultivation practices and inadequate nutrient supply in these two regions. On a dry weight basis, the total phenolic contents observed in this study were within the range of previous mulberry studies [38]. However, the total flavonoid contents were higher, possibly due to the distinctive climatic conditions of Xinjiang.

### 3.4. Antioxidant Activity, Enzyme Inhibitory Capacity, and Correlation Analysis

Certain bioactive compounds in mulberry fruits—such as polysaccharides, anthocyanins, flavonoids, and flavanols—exert inhibitory effects on the activities of α-amylase, α-glucosidase, and pancreatic lipase [39]. α-Amylase catalyzes the hydrolysis of glycogen and starch into glucose, while α-glucosidase hydrolyzes glucosidic bonds to release glucose [40]. Inhibiting these enzymes can reduce intestinal glucose absorption, thereby contributing to effective glycemic control [41]. Pancreatic lipase plays a key role in human lipid metabolism; its inhibition reduces lipid absorption and subsequent fat accumulation, which is important for maintaining metabolic health [42]. The inhibitory effects of mulberry fruit extracts (0.3 mg/mL) from the four production regions on α-amylase, α-glucosidase, and lipase activities are shown in Figure 2D. Overall, the extracts exhibited strong enzyme inhibitory activity, indicating a certain hypoglycemic potential. Sample T displayed the strongest α-amylase inhibition among the four samples, with an inhibition rate of 82.50%, but showed relatively weak pancreatic lipase inhibition (50.06%). Sample B exhibited the weakest α-amylase inhibition (53.66%) but the strongest α-glucosidase inhibition (83.88%). Sample H demonstrated the highest pancreatic lipase inhibition (85.44%) but the lowest α-glucosidase inhibition (47.10%). Sample K showed moderate inhibition across all three enzymes, with inhibition rates of 60.04%, 63.23%, and 56.93% for α-amylase, α-glucosidase, and pancreatic lipase, respectively. Compared to freeze-dried blackberry fruit extracts (α-amylase IC_50_: 4.02–7.66 mg/mL; α-glucosidase IC_50_: 0.27–4.09 mg/mL), black mulberries showed greater hypoglycemic potential, especially T and B, whereas H exhibited better lipid-lowering potential. Correlation analysis (Figure 2F) indicated that total phenolics and total anthocyanins were strongly positively correlated with lipase inhibition (*p* < 0.001), while total flavonoids and flavan-3-ols were strongly positively correlated with α-amylase inhibition (*p* < 0.001). Total tannins also contributed to α-amylase inhibition (*p* < 0.05). Notably, no significant correlation was found between phenolic compounds and α-glucosidase inhibition, suggesting that other components (e.g., polysaccharides, alkaloids) may play a more dominant role, or that multiple compounds act synergistically to achieve α-glucosidase inhibition.

Small berries—including grapes, blackberries, blueberries, and mulberries—are rich in phenolic compounds and other bioactive constituents, contributing to their strong antioxidant potential and important health benefits, such as anti-inflammatory, antidiabetic, neuroprotective, and cardioprotective properties [43]. Figure 2E presents the ABTS^+^, DPPH, and hydroxyl radical scavenging activities of mulberries from four regions. At 0.3 mg/mL, samples from B, H, and K showed the highest ABTS^+^ scavenging rates (88.04%, 88.37%, and 89.94%, respectively), whereas T had the lowest (80.57%). DPPH scavenging activity was similar between B (78.15%) and H (80.18%), but both were markedly different from T (65.74%) and K (63.64%). Compared to goji berry extracts (ABTS^+^ IC_50_: 0.195–0.385 mg/mL; DPPH IC_50_: 0.795–1.085 mg/mL), black mulberries exhibited superior antioxidant potential [44]. Hydroxyl radical scavenging activity varied significantly among the four samples, following the order: H (89.33%) > B (86.34%) > T (82.26%) > K (77.97%). It has been previously reported that black mulberries (MY) possess stronger •OH radical scavenging activity than white mulberries (BY). Our results demonstrate that regional factors also cause marked variations in mulberry antioxidant capacity [45]. Correlation analysis (Figure 2F) showed that total phenolics and total anthocyanins were significantly positively correlated with both DPPH and •OH scavenging rates (*p* < 0.001). Total tannins were strongly correlated with •OH scavenging (*p* < 0.01) and also contributed to DPPH scavenging (*p* < 0.05). As reported earlier, phenolic compounds from natural plant sources are efficient natural antioxidants that help protect the body from oxidative stress injury [46]. Furthermore, Saša et al. found that total phenolics in mulberries play a major role in DPPH radical scavenging, which is consistent with the results of the present study [47].

### 3.5. Analysis of Anthocyanin Metabolites in Mulberry Fruits

**Figure 3:**
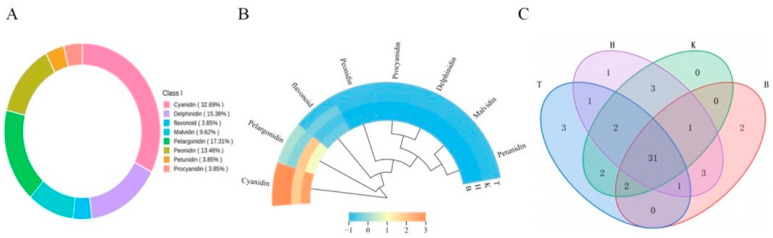
Anthocyanin metabolites in mulberry fruits. Note: (**A**) Anthocyanin metabolite composition; (**B**) contribution of anthocyanin metabolites; (**C**) metabolite Venn diagram.

Anthocyanins are essential bioactive components in mulberry fruits, contributing to environmental stress adaptation and exerting significant health-promoting effects in humans [48]. A total of 52 anthocyanin-related metabolites were identified across the four mulberry samples, primarily comprising cyanidin (17 compounds), delphinidin (8), malvidin (5), pelargonidin (9), peonidin (7), and petunidin (2), along with two flavonoids and two proanthocyanidins. Cyanidin derivatives were the most abundant, accounting for 32.69% of the total anthocyanins, followed by pelargonidin derivatives at 17.31% (Figure 3A). Most anthocyanin metabolites were present in glycosylated forms, predominantly as monoglycosides—such as Cyanidin-3-O-glucoside, Pelargonidin-3-O-glucoside, Cyanidin-3-O-rutinoside, and Peonidin-3-O-glucoside—with a smaller proportion occurring as diglycosides, including Cyanidin-3-O-sambubioside, Pelargonidin-3,5-O-diglucoside, and Delphinidin-3-O-rutinoside-5-O-glucoside. This glycosylation pattern is influenced by growing conditions, environmental factors, and metabolic regulation in the fruit. The type of glycoside affects both metabolism and bioavailability: monoglycosides can be directly metabolized or absorbed via enzymes or intestinal transporters, whereas diglycosides typically require gut microbiota-mediated hydrolysis prior to absorption, leading to more sustained biological effects. The relative contribution of different anthocyanin classes to pigmentation varied by region (Figure 3B). Cyanidin glycosides were particularly abundant in T and K samples, serving as the primary color determinants and directly influencing the purplish-red to black-purple hue of the fruit. This accumulation is likely attributable to intense solar radiation during fruit development in the Turpan and Kashgar regions, which may induce the expression of anthocyanin biosynthetic genes. Pelargonidin derivatives were more pronounced in H, followed by B, contributing stronger reddish tones, potentially influenced by regional temperature regimes. As illustrated in Figure 3C, the four regional samples exhibited distinct anthocyanin metabolite profiles, with 31 compounds commonly shared. Notably, T contained three unique metabolites—Malvidin-3-O-(6″-O-caffeoyl) glucoside, Petunidin-glucoside-galactoside, and Petunidin-3-O-(6″-O-caffeoyl) rhamnoside—which may enhance reddish and bluish-violet coloration while conferring superior stability and antioxidant capacity due to their structural features. B samples uniquely contained Cyanidin-3-O-(6″-caffeoyl) rhamnoside, known for its high stability and antioxidant potential, and Malvidin-3-O-feruloyl-galactoside, which exhibits enhanced structural stability due to the dual stabilizing effects of methoxylation and phenolic acylation, albeit with relatively reduced antioxidant activity due to the electron-donating nature of methoxy groups. H exclusively contained Delphinidin-3-O-(6-O-malonyl-β-D-glucoside), a characteristic pigment associated with bluish-purple coloration. The acylated structure confers high stability, while the polyhydroxylated configuration provides strong antioxidant potential. These unique metabolites, together with other differentially accumulated anthocyanins, collectively contribute to the regional variations in fruit coloration and bioactivity observed among the four black mulberry populations.

**Figure 4:**
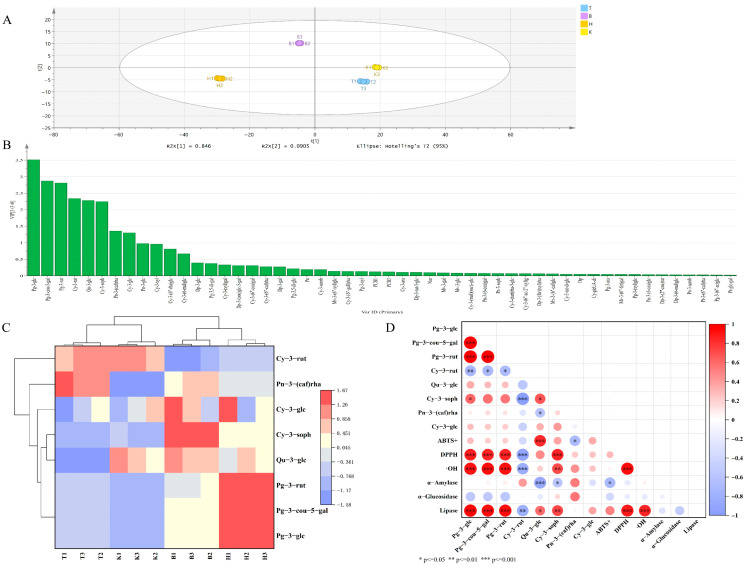
Screening of differential anthocyanin metabolites among mulberry fruits from four production regions. Note: (**A**) OPLS−DA score Plot; (**B**) 200 permutation tests; (**C**) differential metabolite heatmap; (**D**) correlation analysis.

To conduct a differential analysis of anthocyanin metabolites among mulberry fruits from the four regions, OPLS-DA was applied for metabolite selection and discrimination (Figure 4A). Model stability was assessed using sevenfold cross-validation, and the CV-ANOVA test showed “*p* < 0.001”. The reliability of the model was further validated by 200 permutation tests, with all samples within the 95% confidence interval. The model parameters (R^2^X = 0.999, R^2^Y = 0.999, Q^2^ = 0.996) indicated strong explanatory and predictive ability. Moreover, the minimal difference between Q^2^ and R^2^Y confirmed that the model was reliable and free from overfitting. The score plot revealed distinct separation among the four groups, with tight intra-group clustering and clear inter-group differentiation, demonstrating that anthocyanin metabolite profiles are region-specific. The robustness of the model enabled the screening of differential metabolites based on variable importance in projection (VIP) values (Figure 4B). Using a VIP threshold greater than 1, a total of eight key differential metabolites were identified, primarily comprising anthocyanidins and flavonoids. The correlation heatmap of these metabolites is presented in Figure 4C. Overall, the anthocyanin profiles of T and K samples were more similar to each other, whereas greater dissimilarities were observed between these two regions and B and H. This pattern may be attributed to the longer sunshine duration and higher temperatures during fruit development in Turpan and Kashgar. Pelargonidin-3-O-glucoside, pelargonidin-3-O-p-coumaroyl-5-O-galactoside, and pelargonidin-3-O-rutinoside were highly accumulated in H samples, with concentrations of 464.30, 298.09, and 279.57 μg/g (FW), respectively. These compounds predominantly influence fruit coloration, imparting a more stable red hue, while also contributing to a certain degree of astringency. In B samples, cyanidin-3-O-glucoside, cyanidin-3-O-sophoroside, and quercetin-3-O-glucoside (isoquercitrin) exhibited strong correlations and high contents, reaching 1602.66, 76.18, and 31.18 μg/g (FW), respectively. The first two compounds are primarily responsible for the purplish coloration of the fruit. Although quercetin-3-O-glucoside does not directly contribute to color, it serves as an important copigment that interacts with anthocyanins via intermolecular forces, thereby enhancing and stabilizing anthocyanin chromogenicity and preventing color degradation. Notably, cyanidin-3-O-glucoside has been reported to exhibit potent antioxidant activity and beneficial bioactivities, including the modulation of gut microbiota and inhibition of tumor cell proliferation [49,50]. Peonidin-3-O-(caffeoyl) rhamnoside, a methylated and acylated derivative of cyanidin, exhibits a deep reddish-purple hue. The caffeoyl modification substantially enhances its photothermal stability, contributing to the maintenance of vibrant fruit coloration. This metabolite was highly expressed in T samples, with a content of 8.19 μg/g (FW). Cyanidin-3-O-rutinoside, a major contributor to purplish-red coloration, was prominently accumulated in the T and K samples, with contents of 306.12 and 308.11 μg/g (FW), respectively. In summary, significant variations were observed in both the composition and concentration of anthocyanin metabolites among mulberry fruits from different production regions. These differences are primarily influenced by fruit growth conditions and intrinsic metabolic regulation, with environmental factors during fruit development playing a critical role in modulating anthocyanin biosynthetic pathways.

The influence of differential anthocyanin metabolites on the antioxidant capacity and enzyme inhibitory activities of mulberry fruits was evaluated using Pearson correlation analysis, with the results presented in Figure 4D. Quercetin-3-O-glucoside (isoquercitrin) exhibited a significant positive correlation with ABTS^+^ radical scavenging activity (*p* < 0.001). In the mulberry fruit matrix, the ABTS^+^ scavenging effect appears to be primarily attributed to isoquercitrin. This may be explained by its flavonol glycoside structure, which enables electron donation to ABTS^+^, forming a relatively stable semiquinone radical intermediate. This intermediate is resistant to structural tautomerism or sharp declines in activity caused by pH fluctuations, thereby ensuring more stable antioxidant contributions under complex matrix conditions [51]. In contrast, quercetin-3-O-glucoside showed a significant negative correlation with α-amylase inhibitory activity (*p* < 0.001), suggesting that other phenolic compounds or polysaccharides may be the dominant contributors to α-amylase inhibition in mulberry fruits. Pelargonidin-3-O-glucoside, pelargonidin-3-O-p-coumaroyl-5-O-galactoside, and pelargonidin-3-O-rutinoside were significantly positively correlated with both DPPH and hydroxyl radical scavenging activities (*p* < 0.001). Cyanidin-3-O-sophoroside also exhibited a significant positive correlation with these two radical scavenging activities (*p* < 0.01). As a core anthocyanidin structure, Pg-3-glc itself serves as an effective antioxidant unit. The introduction of rutinose in Pg-3-rut confers excellent water solubility, which, under the solvent and pH conditions provided by the high-moisture mulberry matrix, facilitates single-electron transfer or hydrogen atom transfer driven by low O-H bond dissociation energy and redox potential, making it a prominent contributor to radical scavenging [52]. Pg-3-cou-5-gal contains both an anthocyanidin core and a p-coumaroyl moiety; the acyl group may synergistically interact with the anthocyanin system through extended conjugation, thereby enhancing radical scavenging activity [53]. Although the correlation of Cy-3-soph with antioxidant activity was slightly weaker than that of the pelargonidin derivatives, it still exhibited strong intrinsic antioxidant capacity due to the presence of ortho-dihydroxyl groups on the B-ring of its cyanidin backbone. Additionally, these four metabolites—Pg-3-glc, Pg-3-rut, Pg-3-cou-5-gal, and Cy-3-soph—showed significant positive correlations with pancreatic lipase inhibitory activity. This suggests that they may interact with lipase through one or more mechanisms, such as competitive binding with the substrate, thereby contributing to the observed lipase inhibition potential. In conclusion, anthocyanidins and flavonoids in mulberry fruits exhibit substantial free radical scavenging capacity and enzyme inhibitory activities, underscoring their potential as antioxidant and hypoglycemic agents. Variations in glycosylation patterns differentially modulate the bioactive contributions of these metabolites, collectively shaping the functional properties of mulberry fruits.

### 3.6. Analysis of Volatile Metabolites and Aroma Profiles in Mulberry Fruits

**Figure 5:**
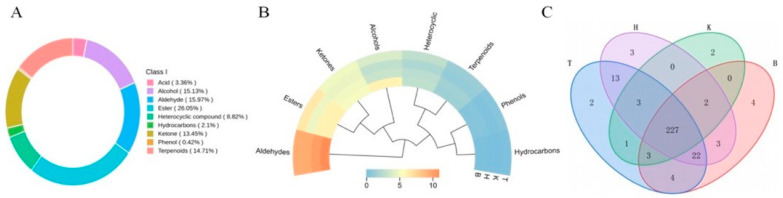
Volatile metabolites in mulberry fruits. Note: (**A**) Classification of volatile substances; (**B**) contribution of volatile substances; (**C**) volatile metabolite Venn diagram.

A total of 292 volatile metabolites were identified across the four mulberry samples using headspace solid-phase microextraction (HS-SPME) coupled with gas chromatography-mass spectrometry (GC-MS). These included 78 esters, 45 alcohols, 12 phenols and acids, 46 aldehydes, 40 ketones, 36 terpenes, 10 hydrocarbons, and 25 heterocyclic compounds. Overall, esters constituted the most abundant class of volatile compounds, accounting for 26.05% of the total volatiles, followed by aldehydes (15.97%), alcohols (15.13%), terpenes (14.71%), and ketones (13.45%). These diverse volatile profiles collectively contribute to the characteristic aroma complexity of mulberry fruits (Figure 5A). The contribution of individual volatile classes to the overall aroma profile varied considerably across production regions, reflecting the influence of growing environment on volatile biosynthesis (Figure 5B). Aldehydes were the predominant contributors to aroma in all four regional samples. Notably, T samples exhibited a higher relative abundance of esters compared to the other three regions, imparting more pronounced fruity and winy notes, which may be associated with differences in fruit maturity. In contrast, B samples were characterized by higher relative abundances of ketones and alcohols, contributing fruity, floral, citrus, pine, and cooling medicinal-like aromas. Previous research has demonstrated that within the same production region, black mulberries possess a stronger aroma than white mulberries, with aldehydes and esters serving as the main odor contributors. This difference is attributed to genotypic variation among the fruits. In contrast, for the same variety grown in different locations, aroma differences are primarily due to environmental factors that regulate metabolic processes during fruit ripening [54]. To further elucidate differences in volatile composition among the four mulberry fruit types, preliminary screening of unique volatile compounds was conducted (Figure 5C). T samples contained two unique compounds: eugenol, contributing subtle floral and spicy notes, and ethyl laurate, imparting a beeswax-like aroma accompanied by light, sweet fruity undertones. B samples uniquely contained four compounds: 2-propylfuran, characterized by green bean-like and grassy notes; 5-methyl-2-hexanone, exhibiting neutral fruity and grassy aromas; 4-methyl-4-penten-2-one, presenting predominantly sweet–sour fruity notes blended with an unripe fruit freshness; and (Z)-2,3-dimethyl-3-heptene, predominantly grassy and leafy with slight unripe fruity nuances. Collectively, these compounds likely contribute to a fresh, green fruity aroma profile in B samples. H samples uniquely contained three compounds: 6-undecanol, presenting mild, persistent fatty and waxy notes with subtle fresh grassy and citrus peel nuances; ethyl benzoate, exhibiting distinct cherry- and strawberry-like fruity and floral notes; and tetradecane, which contributes minimally to the overall aroma profile. K samples uniquely contained two compounds: methyl 3,4-dimethylbenzoate and 3,3,6-trimethyl-1,5-heptadien-4-ol, both presenting faint sweet, herbal, and woody notes. The presence of these compounds may indicate a decline in fruit freshness, potentially attributable to over-ripening or prolonged postharvest storage.

**Figure 6:**
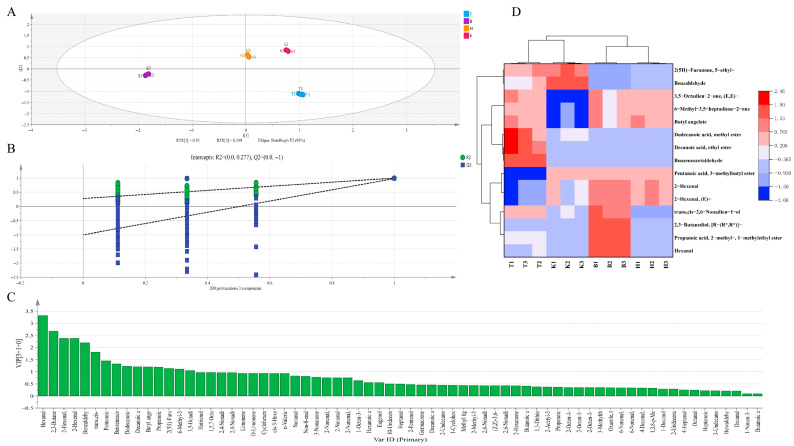
OPLS−DA analysis of volatile metabolites in mulberry fruits. Note: (**A**) OPLS−DA score Plot; (**B**) 200 permutation tests; (**C**) VIP image; (**D**) differential metabolite heatmap.

To identify key differences in volatile compounds among black mulberries from different regions, a second screening based on odor contribution was performed. Sixty-nine compounds with rOAV > 1 were selected and analyzed using OPLS-DA, which effectively distinguished the four mulberry samples (Figure 6A). Model stability was validated by sevenfold cross-validation, and the CV-ANOVA test showed “*p* < 0.001”, with all samples within the 95% confidence interval. The model parameters (R^2^X = 0.98, R^2^Y = 0.996, Q^2^= 0.985) demonstrated strong explanatory and predictive ability. Moreover, the minimal difference between Q^2^ and R^2^Y confirmed that the model was reliable and free from overfitting. Model reliability was further validated through a 200-permutation test (Figure 5B). The score plot revealed significant regional differences in volatile composition among the four production regions. The VIP (variable importance in projection) plot of important volatile metabolites in mulberry fruits from different regions is shown in Figure 6C. Using a VIP threshold greater than 1, a total of 15 key differential metabolites were identified. To more intuitively visualize the differences in these key metabolites, hierarchical clustering analysis was performed, and the resulting heatmap is presented in Figure 6D. The aroma profiles of T and K samples were relatively similar to each other, while both differed considerably from those of B and H. In T samples, the most influential aroma contributors were methyl laurate (mild fruity and floral notes), ethyl decanoate (strong grape-like and winy notes), and phenylacetaldehyde (intense hyacinth-like floral and honey-sweet notes), with relative contents of 0.15, 0.12, and 0.17 μg/g (FW), and rOAV values of 43.39, 23.56, and 27.17, respectively. B samples were characterized by compounds imparting fresh and green notes: hexanal (grassy and leafy), 1-methylethyl 2-methylpropanoate (intense fruity), [R-(R,R)]-2,3-butanediol (faint creamy), and (E,Z)-2,6-nonadien-1-ol (strong fresh cucumber and watermelon rind-like aroma). Their relative contents were 1.10, 0.14, 0.75, and 0.36 μg/g (FW), with rOAV values of 220.57, 5.43, 7.92, and 360.20, respectively. In K samples, 5-ethyl-2(5H)-furanone (strong caramel and baked bread-like aroma) and benzaldehyde (bitter almond, cherry pit-like nutty notes) contributed positively to the aroma profile, with relative contents of 0.13 and 0.17 μg/g (FW) and rOAV values of 23.06 and 1.48, respectively. Compared with the other three regions, H exhibited a more complex aroma profile, likely arising from the synergistic contribution of multiple volatiles. On a fresh weight basis, these included (E)-2-hexenal (green, leafy; 4.04 μg/g, rOAV 1302.24), 2-hexenal (4.04 μg/g, rOAV 237.47), butyl (Z)-2-methyl-2-butenoate (0.48 μg/g, rOAV 36.98), 3-methylbutyl pentanoate (tropical fruity; 0.29 μg/g, rOAV 2.91), (E,E)-3,5-octadien-2-one (woody, spicy; 0.38 μg/g, rOAV 768.41), and 6-methyl-3,5-heptadien-2-one (0.41 μg/g, rOAV 4.15). The contribution of these diverse, plant-like volatiles elevates the black mulberry’s aroma profile from simple sweetness to a flavor experience of “wildness” and “complexity”.

## 4. Conclusions

In black mulberries from four Xinjiang regions—Turpan (T), Bazhou (B), Hotan (H), and Kashgar (K)—fructose and glucose were the dominant sugars, with the highest levels in K and the lowest in B. Organic acids included tartaric, malic, citric, succinic, and quinic acids, with significant regional variation. Citric acid accounted for the largest proportion, whereas tartaric acid was the lowest and showed no regional differences. Due to regional variations in climate and altitude, the phenolic composition and bioactivity of mulberries differed markedly among the four regions. Hotan (H) had the highest total phenolic and total tannin contents, while Turpan (T) had higher total flavonoid and flavan-3-ol contents. Bazhou (B) and Hotan (H) exhibited stronger antioxidant potential. Additionally, T, B, and H showed the strongest inhibition of *α*-amylase, *α*-glucosidase, and lipase, respectively. A total of 52 anthocyanins and related metabolites were identified, with cyanidin (32.69%) and pelargonidin (17.31%) as the major aglycones. Eight key differential metabolites were screened via OPLS-DA, which specifically influenced fruit color (e.g., cyanidin-3-O-rutinoside), stability (e.g., pelargonidin-3-O-glucoside), and antioxidant potential (e.g., cyanidin-3-O-glucoside). Equally important, the aroma composition also varied significantly with region. Among the 292 volatile metabolites detected, 15 (e.g., hexanal, methyl laurate) served as key discriminators. T exhibited more fruity and floral notes, whereas K presented more pronounced stone fruit and roasted aromas. In conclusion, environmental factors such as altitude, temperature, and humidity substantially influence the chemical composition and bioactivity of black mulberries. A systematic understanding of regional fruit characteristics not only aids in evaluating their fresh and processing traits, but also supports the development of region-specific high-value mulberry products. Furthermore, this study provides data-driven insights into flavor quality differentiation and establishes a foundation for optimizing mulberry cultivation practices and achieving the targeted regulation of key compounds responsible for color, aroma, and bioactivity.

## Figures and Tables

**Table 1:** Sampling situation of the four production areas.

Sampling Point	Geographical Location	Topographical Features	Climate Characteristics	Altitude	Annual Average Precipitation	Average Annual Hours of Sunshine	Sampling Details
Turpan Gaochang District Black Mulberry Plantation Base	42° N, 88° E	High in the north and low in the south, extremely low in the center of the basin	Abundant sunlight, ample heat, extremely dry, low precipitation, and frequent strong winds	0–35 m	14.7 mm	2788 h	Base planting, according to the planting row spacing, take a 100 g sample every 10 m
Luntai County, Bazhou	41° N, 84° E	The overall terrain is higher in the north and lower in the south.	Long hours of sunlight, long frost-free period, sparse precipitation, vigorous evaporation, dry air	900–1100 m	89 mm	2658 h	Non-standardized planting, sample 100 g at 2 km intervals
Hotan City Sericulture Science Research Institute	37° N, 79° E	Slightly sloping from south to north, with a very gentle gradient	The climate is dry, with low precipitation and abundant sunshine throughout the year	1300–1400 m	36.4 mm	2644 h	Sample at 10 m intervals according to the planting row spacing
Yingjisha County, Kashgar	38° N, 76° E	Overall flat and open	Small interannual temperature variation, large day-night temperature difference, little precipitation	1250–1300 m	81.5 mm	2638 h	Non-standardized planting, sample 100 g at 2 km intervals

Chemicals: Gallic acid, rutin, (+)-catechin, 2,2-diphenyl-1-picrylhydrazyl (DPPH), 2,2′-azino-bis(3-ethylbenzothiazoline-6-sulfonic acid) (ABTS), α-amylase (14 U/mg), α-glucosidase (50 U/mg), porcine pancreatic lipase (15–35 U/mg), p-hydroxybenzoic acid hydrazide (PAHBAH), 4-nitrophenyl-β-D-glucopyranoside, and p-nitrophenyl laurate were purchased from Source Leaf Biotechnology Co. (Shanghai, China).

**Table 2:** Basic physico-chemical properties of black mulberry fruits from different producing areas.

	T	B	H	K
Fresh Weight of Fruit (g)	2.28 ± 0.49 c	5.89 ± 2.09 a	4.19 ± 0.72 b	1.95 ± 0.69 c
V (cm^3^)	2.65 ± 0.76 c	5.50 ± 1.48 a	3.90 ± 0.85 b	1.94 ± 0.37 c
Fresh Weight of Twenty Fruits (g)	50.7 ± 5.63 c	119.30 ± 1.31 a	84.24 ± 0.61 b	39.73 ± 0.60 d
Dry Weight of Twenty Fruits (g)	6.48 ± 1.31 c	12.08 ± 0.68 a	9.29 ± 0.17 b	10.63 ± 0.32 ab
Longitudinal Diameter (mm)	31.36 ± 4.26 b	37.67 ± 4.72 a	29.82 ± 3.80 b	19.25 ± 3.19 c
Transverse Diameter (mm)	13.74 ± 1.30 b	16.40 ± 2.08 a	13.30 ± 1.47 b	10.96 ± 1.83 c
Stem Length (mm)	4.91 ± 1.57 c	6.03 ± 2.05 bc	9.97 ± 1.80 a	7.23 ± 1.45 b
Fruit Shape Index	2.28	2.3	2.25	1.78
L*	57.41 ± 0.85 b	60.20 ± 2.04 a	73.33 ± 2.68 ab	67.16 ± 2.35 b
a*	12.07 ± 1.82 b	17.87 ± 3.56 a	15.73 ± 5.60 ab	13.47 ± 3.38 b
b*	−18.38 ± 2.71 a	−27.85 ± 7.77 b	−22.44 ± 9.93 ab	−23.15 ± 7.93 ab
Soluble Solids Content (°Brix)	17.68 ± 2.50 a	9.71 ± 1.57 c	13.99 ± 1.02 b	18.88 ± 2.05 a
Reducing Sugar (FW g/L)	118.00 ± 2.29 b	42.00 ± 1.20 c	50.80 ± 4.21 c	144.00 ± 10.82 a
Titratable Acidity (FW g/L)	4.29 ± 0.12 c	5.31 ± 0.05 b	8.04 ± 0.04 a	2.96 ± 0.08 d
pH	4.83 ± 0.06 b	3.63 ± 0.06 c	3.47 ± 0.06 d	5.47 ± 0.06 a

Note: The black mulberry samples from the four production areas of ‘Turpan’, ‘Bazhou’, ‘Hotan’, and ‘Kashgar’ are respectively named T, B, H, and K. Values in a column followed by a different letter are significantly different according to Duncan’s multiple range test at *p* < 0.05 level.

## Data Availability

The original contributions presented in this study are included in the article. Further inquiries can be directed to the corresponding authors.
